# ﻿Phototank setup and focus stack imaging method for reptile and amphibian specimens (Amphibia, Reptilia)

**DOI:** 10.3897/zookeys.1134.96103

**Published:** 2022-12-09

**Authors:** Emily M. Braker

**Affiliations:** 1 Vertebrate Zoology, University of Colorado Museum of Natural History, UCB 265, Boulder CO 80309, USA University of Colorado Museum of Natural History Boulder United States of America

**Keywords:** digitization, focus stack photography, Helicon Focus, herpetology collections, imaging, phototank

## Abstract

Fluid-preserved reptile and amphibian specimens are challenging to photograph with traditional methods due to their complex three-dimensional forms and reflective surfaces when removed from solution. An effective approach to counteract these issues involves combining focus stack photography with the use of a photo immersion tank. Imaging specimens beneath a layer of preservative fluid eliminates glare and risk of specimen desiccation, while focus stacking produces sharp detail through merging multiple photographs taken at successive focal steps to create a composite image with an extended depth of field. This paper describes the wet imaging components and focus stack photography workflow developed while conducting a large-scale digitization project for targeted reptile and amphibian specimens housed in the University of Colorado Museum of Natural History Herpetology Collection. This methodology can be implemented in other collections settings and adapted for use with fluid-preserved specimen types across the Tree of Life to generate high-quality, taxonomically informative images for use in documenting biodiversity, remote examination of fine traits, inclusion in publications, and educational applications.

## ﻿Introduction

Biological collections contribute deep reservoirs of anatomical and morphological information for extinct and extant biodiversity and are essential for our understanding of life on Earth. While molecular approaches are now a central means for delimiting species and understanding phylogenetic relationships, characterization of the phenotype remains fundamental to analyzing patterns of diversity across space and time (Wiens 2008; Wake 2012; Lee and Palci 2015). Newly developed technologies and cyberinfrastructure improvements to capture, store, and disseminate phenotypic data have the potential to accelerate morphological research across wide-ranging disciplines such as evolutionary biology, ecology, and conservation science. Central to facilitating increased access to morphological information is the translation of physical specimen resources into digital datasets and products, which has increasingly become a core role of natural history collections in the 21^st^ century.

Paralleling the global sea change from analog to digital technologies, the past two decades have witnessed a major shift in the availability of natural history data through the mass digitization of collections and associated archives. Initiatives and funding efforts, such as the US National Science Foundation’s (NSF: http://nsf.org) Advancing Digitization of Biological Collections and Infrastructure Capacity for Biology programs (both replaced with the Infrastructure Capacity for Biological Research program in 2020), have mobilized collections to publish taxonomic, geographic, temporal, and morphological data at unprecedented scales, expanding the traditional reach of museums, and inviting participation of new research communities and downstream users through enabling widespread data sharing and opportunities for collaboration (Blagoderov et al. 2012; Nelson and Ellis 2019; Hedrick et al. 2020; Hilton et al. 2021). While voucher specimens remain the gold standard format for archiving biodiversity and conferring repeatability in scientific studies, digital products such as two-dimensional images and computed tomography (CT) media serve as extensions of physical collections and add value and utility to preserved specimens (Beaman and Cellinese 2012; Webster 2017; Hedrick et al. 2020; Lendemer et al. 2020; Hilton et al. 2021). Such digital proxies also play a vital role in the long-term preservation of primary resources, at times circumventing the need to loan, handle, or dissect specimens, thereby reducing risks to physical collections (Blagoderov et al. 2012; Brecko et al. 2014; Page et al. 2015; Lendemer et al. 2020).

Furthermore, building online digital media repositories is a democratizing force in promoting collections access (Boyer et al. 2016; Hedrick et al. 2020; National Academies of Sciences, Engineering, and Medicine 2020). Conducting research visits to museums, field stations, or other biological archives to examine specimens often requires significant budgetary and time investments (Page et al. 2015; Kaiser et al. 2018), limiting widespread participation and presenting major impediments to international collaborations. While specimen loans are typically less resource-intensive than coordinating collections visits, accessibility issues are still present. Specimen loan volumes are generally limited to what is deemed a reasonable quantity for collections staff to prepare and ship, with in-house examination highly encouraged for large sample sizes. Concurrent borrowing of the same physical specimen by more than one researcher is not possible, and often investigators must wait the full duration of a loan period (typically 6–24 months) for return of a needed individual(s) before it is eligible for use in their own project. Wait times are also extended by the common museum practice of loaning out no more than one half of a given taxonomic series as a safeguard against loss, whereby investigators borrow requested material in multiple loan installments, returning each batch before the next is processed. Protective museum loan policies may necessarily circumscribe collections accessibility, often prohibiting shipments of type specimens, endangered species, fragile material, and rare series, or restricting loans to countries where wildlife shipments are viewed as overly risky or administratively burdensome. Conversely, online digital formats are free of such constraints and may expedite research timelines when specimen surrogates are suitable for use. Similarly, specimen media offer an alternative research modality when collections or loan access is disrupted by events such as natural disasters or infectious disease outbreaks, as experienced during widespread and enduring operational shutdowns amidst the COVID-19 pandemic.

Specimen images are a particularly effective tool in that they facilitate curatorial, research, and educational enterprises. For instance, photographs provide a timestamped snapshot that conveys both specimen disposition status and condition, aiding in collections security, inventory control, and assessment (Blagoderov et al. 2012). Risk of catastrophic damage and loss from natural disasters, failing infrastructure, and housing highly flammable collections is an unfortunate reality for natural history institutions (e.g., Butantan Institute and National Museum of Brazil fires in 1978 and 2018, respectively), and digitization provides an alternative preservation mechanism to virtually document and depict collections materials and ultimately sustain their utility in the case of destruction. Photographs can also be used to initially evaluate the suitability of physical specimens for research or loan (Kaiser et al. 2018), economizing resources and reducing unnecessary borrowing.

In a research context, photographs play an essential role in documenting biodiversity (Mertens et al. 2017; Lunghi et al. 2020), and baseline imagery is especially essential for conservation managers and wildlife biologists working with rare or cryptic species known to science by only a few individuals or accounts. High-quality images can enable verification of taxonomic identifications (Ariño and Galicia 2005; Wheeler et al. 2012) and support sex determinations in dimorphic species, and images are increasingly requested by researchers in lieu of physical specimen loans when diagnostic traits can be observed in a two-dimensional format. Specimens figured in publications enhance textual descriptions and are key elements for communicating morphologically representative traits or novel research concepts. Critically, high-resolution photographs provide raw trait data to be extracted and analyzed for any number of phenomic applications, such as investigations in comparative morphology, hybridism, and pattern morphs (Dittmer et al. 2015), landmark-based geometric morphometrics (Muir et al. 2012), and training convolutional neural networks in image recognition and classification (Nelson and Ellis 2019; Hedrick et al. 2020; Soltis et al. 2020; Durso et al. 2021). As new analytical tools are developed, the greater the potential for automation of rote tasks and meristic data mining from images such as scale counts and character scoring (Ziegler et al. 2010), high-throughput phenotyping, and greater yields in morphological data to advance biodiversity research, engage citizen scientists, and guide agency-based wildlife management practices (Chang and Alfaro 2016; Hedrick et al. 2020; Medina et al. 2020).

Finally, specimen images are broadly useful for public audiences, from incorporation in museum exhibitions to subject reference for field guide illustrations and artwork. Increasingly, mobilized biodiversity data, including images, are used to enrich STEM curricula in primary, secondary, and university education, including online learning environments. Integration of digitized collections in education promotes active, inquiry-based learning in core biological concepts, bolstering scientific literacy and providing engaging and transformative experiences to inspire the next generation of biodiversity scientists (Cook et al. 2014; Powers et al. 2014; Monfils et al. 2017; Ellwood et al. 2020).

## ﻿Imaging challenges

It is estimated that only 10% of biological collections data are available online of the estimated one billion specimens housed in US institutions (Page et al. 2015). More limited still is the availability of trait and morphological data, which are essential to the interpretation of the fossil record and investigations into biological and ecological processes such as adaptation, community assemblage, evolutionary convergence and divergence, and speciation (Mayr 1956; Winker 2009; Mahler et al. 2013). Challenges and bottlenecks related to specimen imaging methods likely foster the gap in phenomic data, particularly for groups with complex three-dimensional forms. This is evidenced by the overwhelming dominance of plant specimen imagery in the biodiversity media landscape, with the relatively flat herbarium sheet format more compatible with high-throughput capture methods and mass-digitization than other specimen preparation types common within zoological and paleontological collections (Baker 2011; Ball-Damerow et al. 2019). Vertebrate groups are especially poorly represented, comprising just 3.3% of all image media linked to preserved specimens on the Global Biodiversity Information Facility (GBIF: http://www.gbif.org; GBIF 2022a). In particular, species-diverse clades such as fishes, amphibians, and reptiles that collectively comprise more than 80% of vertebrate diversity make up the smallest proportion of vertebrate specimen images on GBIF (1.1%). This is almost certainly due to the standard preparation convention of fluid-preservation in these groups, which brings its own suite of imaging challenges, including glare and reflectance when removing specimens from storage solution (Sabaj 2008; Kaiser et al. 2018), and the risk of dehydration and damage to specimens when photographed outside of a wet environment.

Reptile and amphibian specimens present specific imaging challenges. Unlike the majority of fishes which share a relatively flat, compressed body plan that is conventionally photographed from a lateral aspect, reptiles and amphibians minimally necessitate dorsal and ventral views to comprehensively observe morphology. Diagnostic features such as scale shape, arrangement, texture, and patterning typically require high-resolution images and zoom magnification in order to adequately examine and quantify traits. Spiny projections and textured skin topography, significant size variation, and specimens with tall profiles such as turtles and coiled snakes can add considerable depth to images, creating out-of-focus regions within the composition. Poorly prepared specimens in nonstandard positions are also commonplace in natural history collections containing historic material. For instance, specimens fixed without use of a hardening tray and directly immersed in formalin as a method of euthanasia (a now outmoded practice) tend to be contorted instead of neatly coiled or with limbs or tails squarely posed in a flat plane, making them difficult to position and sharply render each body element in photographs. These collective issues very likely contribute to the paucity of reptile and amphibian specimen images available online and the overall lack of concerted digitization programs that emphasize fluid-prepared herpetofauna (Longson et al. 2018; Brecko and Mathys 2020). The vast majority of the 273,657 herpetology specimen images available on GBIF are comprised of specimens photographed while being processed during fieldwork (GBIF 2022b, c). While preserving color immediately following death, these images vary greatly in terms of standardization and quality.

**Figure 1.**
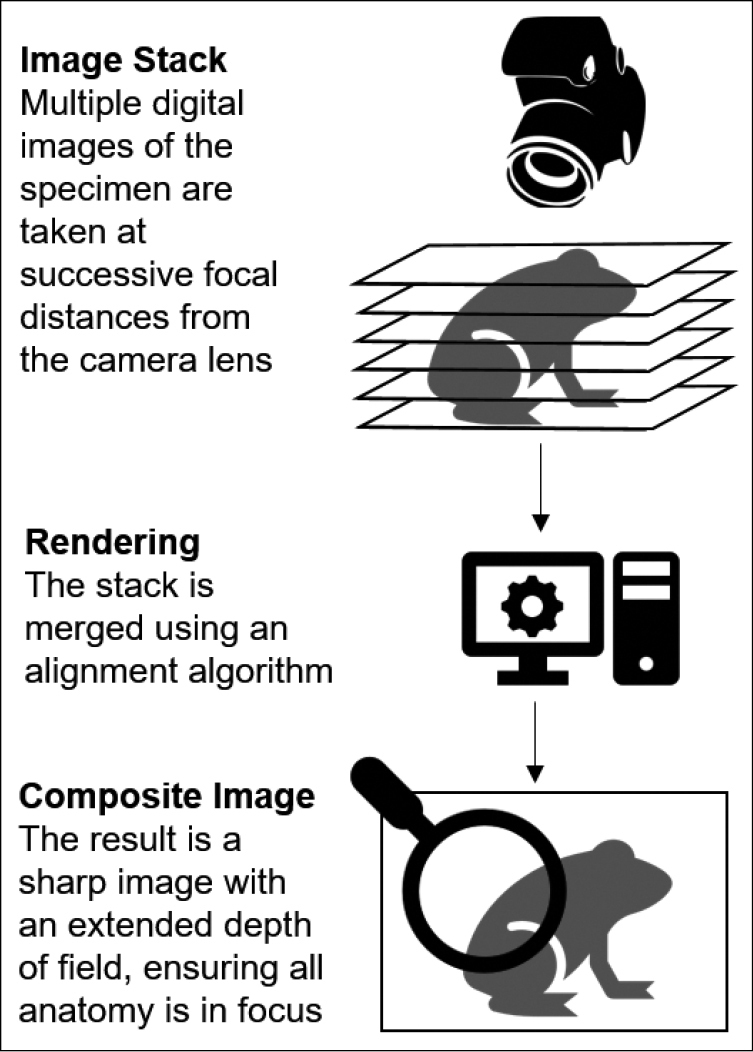
Focus stacking process overview.

Two approaches that counteract these imaging complexities include focus stack photography and the use of a photo immersion tank (phototank) to image specimens. Focus stack photography (also known as Z-stacking) involves taking several images of a subject at successive focal distances that are then merged to create an image with an extended depth of field (Fig. 1). This method requires mathematical processing to combine the source images into a single composite photo that is entirely in focus. Focus stack photography has been extensively used by the entomology and macroscopy communities (Mertens et al. 2017; Longson et al. 2018) and to a lesser extent for imaging dry vertebrate material such as skulls and study skins (Nelson et al. 2012). This method produces exceptional quality research-grade images that enable close examination of fine traits.

**Figure 2.**
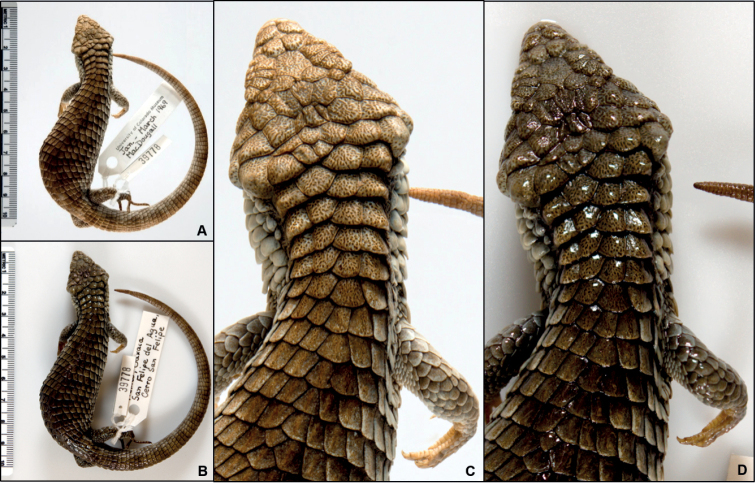
UCM 39778*Abroniaoaxacae*, focus stacked images under the same lighting conditions using **A** immersion in a phototank versus **B** dry photography methods. Enlarging these photographs illustrates **C** the greater legibility in scale patterning with the wet setup, while **D** glare, shadow, and a darker cast are produced when the specimen is removed from preservative for imaging.

Employing a phototank to immerse specimens in preservative during imaging eliminates reflection interference associated with dry imaging methods (Randall 1961; Emery and Winterbottom 1980; Sabaj 2008; Kaiser et al. 2018). A preservative bath also provides physical support and maintains specimen hydration, and better reproduces patterning in images, which tends to be darker and more difficult to see outside of fluid (Fig. 2). Imaging “squeeze tanks,” initially developed for photographing live fish and later adopted in the digitization era for imaging preserved specimens, have been in use by the ichthyology collections community for decades (Randall 1961; Emery and Winterbottom 1980; Holm 1989; Sabaj 2008). Though photographing anesthetized salamanders under water has been documented at least once (Lanza et al. 1995), given the limited application of squeeze tanks with live herpetofauna, particularly with fully terrestrial species, a parallel technology transfer to specimen-based photography has not occurred within herpetology. Time and staffing constraints may further contribute to the relative lack of wet photography of fluid-preserved reptile and amphibian specimens.

## ﻿A combined method for imaging reptile and amphibian specimens

**Figure 3.**
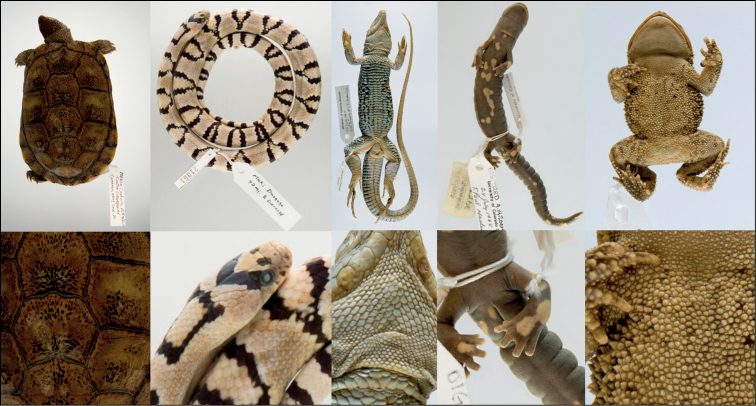
Image gallery of selected specimens from the University of Colorado Museum of Natural History (UCM) Herpetology Collection produced using a combined focus stack photography and phototank methodology. The bottom row reveals the morphological detail captured with this modality using zoom magnification. Left to right: UCM 48846*Terrapenecoahuila*, UCM 21061*Lampropeltismexicanagreeri*, UCM 35425*Aspidoscelisstictogrammus*, UCM 25520*Bolitoglossalincolni*, UCM 41256*Inciliuscycladen*.

The following methods detail a procedure for combining focus stacking and wet photography techniques used by the University of Colorado Museum of Natural History (**UCM**) as part of an NSF-funded digitization project to create high quality squamate and amphibian specimen images (NSF #2001474 oMeso: Opening Mesoamerican Herpetofaunal Diversity to Whole Phenome Imaging [oMeso]; Fig. 3). Equipment, workflow, and recommendations are provided as a roadmap for implementing this approach in other collections settings, with the opportunity to modify the system to accommodate fluid-preserved specimen types across the Tree of Life.

### ﻿Equipment

**Figure 4.**
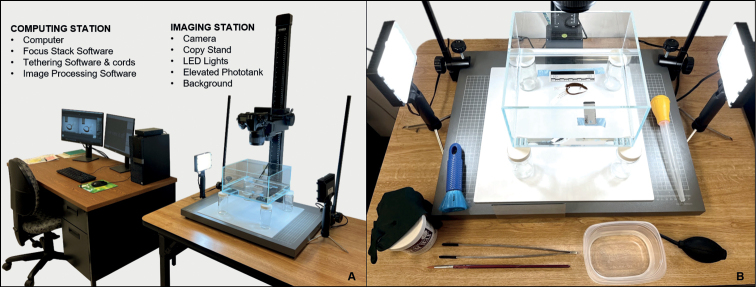
UCM equipment components and configuration. **A** the computing and imaging stations are physically separated to avoid liquid damage **B** detail of phototank setup and accessories with specimen positioned for imaging (shallow aquarium filled with ethanol, scale bar, white balance card, masking tape, jars, acrylic background, flocked nitrile gloves, wax, duster, coated forceps, paintbrush, rinse container, air dust blower, bulb syringe).

This methodology requires three basic components: (i) photography equipment, (ii) photo immersion tank setup and supplies, and (iii) focus stack imaging software and accessories (Fig. 4, Table 1). While specific brands used by the UCM Herpetology Collection are noted in this section, much is possible in the way of substitution and improvisation.

**Table 1.** Recommended equipment list summary.

Phototank and focus stack photography equipment list	
**Camera equipment**	**Remarks**
Camera body	Professional grade DSLR
Lens	Recommended 50–100 mm
Copy stand	High stability with arm length dependent on maximum specimen size
Studio lights with diffusers	LEDs preferred if using tabletop or copy stand attachments near tank
Backdrop	Neutral, non-reflective acrylic, blotting paper, etc.
Scalebar	
White balance card	
Air dust blower	Camera lens maintenance
**Phototank and accessories**	R**emarks**
Phototank	Glass adhered with silicone or prefabricated rimless, shallow aquarium
Supports/base	Custom-built frame or improvised supports, e.g., glass jars
Forceps	Silicon-coated for cushion/scratch prevention
Static duster	
Bulb syringe	
Paintbrush	Useful for positioning specimen, tags, and popping bubbles
Lab tape/masking tape	Used for affixing calibration tools to bottom of tank
Preservative	
Small Container	Pre-imaging bath - size dependent on maximum specimen size
Gloves	Recommend flocked nitrile for ease of reuse
Wax/mount	Wax/custom mount for supporting specimens (as needed)
Glass plate	Multiple sizes for flattening tags/specimens (as needed)
**Software and cords**	R**emarks**
Focus Stacking Software	Recommended Helicon Focus or Zerene Stacker
Tethering Software	Compatible with camera model
Power adapter	Supply kit compatible with camera model
Tethering cords	USB compatible with camera model, at least 1.5 m

### ﻿Photography equipment and supplies

#### Camera

A digital single-lens reflex (DSLR) camera body provides dynamic range, high fidelity image detail and ISO performance, as well as versatility in exchangeable lens options. A Nikon D810 camera was used for capturing project specimens (now succeeded by the Nikon D850 model), though any modern DSLR system sourced from a major camera brand such as Canon, Fuji, Nikon, or Sony will reliably produce high-quality images.

#### Lens

A Nikon AF-S Micro-NIKKOR 60 mm f/2.8G ED lens was used to capture project specimens and can approach or achieve a 1:1 magnification ratio or greater for small-bodied specimens. Because reptile and amphibian subjects have wide-ranging body sizes, a 50–100 mm lens is recommended for capturing herpetological specimens with a wet imaging station setup.

#### Copy stand

A copy stand is necessary to securely suspend the camera over the photo tank. A mid-range or high-end option is ideal for mitigating vibrations in the immediate studio facility as well as camera movement when focusing or making fine adjustments to the camera height along the rail. A Kaiser RS10 copy stand with 40” arm was selected for its flexibility in accommodating both extremes of the size spectrum for the oMeso project, from miniaturized salamanders (e.g., *Thorius*, total length ca. 2 cm) to large iguanids (e.g., *Ctenosaura*, ca. 33 cm when prepared in a curled format).

#### Lighting

Many lighting and diffusing options are commercially available that provide flat, even specimen illumination. Low-budget tabletop flat panel LEDs with a diffuser filter (EMART 60 LED Continuous Portable Photography Lighting Kit) were selected for the UCM phototank setup to minimize the potential for fire danger with ethanol. If necessary, a velvet drape or piece of black cardstock with a hole cut in the middle to fit over the camera lens can be used to block reflections from overhead lighting in the tank preservative.

#### Backdrop

A matte white acrylic board (AbleDIY Non-Reflective Acrylic Display Board) placed on the copy stand base was used as an image background. A neutral (white, grey, black), non-reflective backdrop is recommended for overall image legibility and contrast with specimens, and simple solutions such as a sheet of blotting paper or velvet cloth are also appropriate.

#### Calibration tools

A scale bar and white balance card (WhiBal G7) were included as standards for all project images. A physical reference ruler is necessary for calibration purposes even if a digital scale bar is to be inserted into final images. A white balance card is used as a standard to neutralize color casts when processing images. While indoor lighting conditions are far less variable than natural lighting, the color temperature of artificial lights as well as any position adjustments to studio lights between specimens necessitate calibration of each image or photo batch. The scale bar and white balance card were positioned at the periphery of the compositional frame so that they could be easily cropped from final images if desired (e.g., for use in publication figures or online exhibits). For the oMeso project, calibration tools were affixed to the outer surface of the bottom of the phototank with masking tape for ease of repositioning according to individual specimen size. It is worth nothing that a color calibration standard was not included in this project given the known effects of formalin-fixation on specimen pigmentation, which causes significant alteration in hues such as reds, yellows, and greens (Simmons 2014). However, use of a color balance chart is highly encouraged when imaging recently deceased animals or shortly following specimen processing when coloration is still true-to-life.

### ﻿Photo immersion tank and accessories

#### Photo immersion tank and base

Two shallow, rimless aquaria were purchased to carry out digitization (Ultum Nature Systems model 25S, 25.0 × 25.0 × 12.5 cm; model 45S, 45.0 × 28.0 × 18.0 cm). In-house construction of a phototank system is also possible using five panes of glass adhered with silicone. Tank dimensions fit within the footprint of the copy stand baseboard, with relatively short wall height specifications to prevent interference from reflections or shadows on the surface of the bath while still accommodating sufficient preservative volume to fully immerse target specimens during imaging. Whenever possible, the smaller tank size was used in order to minimize ethanol replacement costs throughout the duration of the project. This tank fits the vast majority of squamate and amphibian specimens submerged in approximately 5–10 cm of ethanol, while the larger tank was used to image oversized taxa such as iguanids and varanids, or those with tall profiles, such as turtles and coiled snakes up to 15 cm in height. Jar supports were used to elevate the tank from the copy stand baseboard in order to achieve *bokeh*, a slightly blurred, soft backdrop. Tanks placed directly in contact with a background surface produce a small zone of mirroring around specimens and tend to trap dust and microfibers that require processing out of final images. An elevated tank also allows for backlighting to reduce specimen shadows in images. A custom base frame or supports may be constructed from any number of materials, with clear acrylic recommended as an inconspicuous option. Jars offer a simple solution (Fig. 3), though a frame or supports with a sleeker profile will minimize encroachment into the useable field of view. It is worth noting that it is entirely possible to photograph reptile and amphibian specimens with a traditional squeeze tank setup as is frequently employed in ichthyology collections. This method utilizes a narrow, vertically oriented aquarium paired with a tripod-mounted camera and an angled pane of glass to suspend the specimen in the middle of the tank during imaging. This approach has the advantage of allowing fine particles and debris to fall out of the field of view to the bottom of the tank, reducing spot-cleaning during the specimen staging and image retouching phases. However, friction-pinning specimens in this position can be challenging and time-consuming and is not always possible across different taxa, body plans, and preparations. Additionally, tripods are less stable than a copy stand configuration and may be more prone to introducing vibration artifacts into Z-stacked media.

#### Preservative

Fresh ethanol (70% concentration) was used to shallowly immerse project specimens during imaging, minimally creating a 5–10 mm layer above each individual’s tallest anatomical feature. While it may be tempting to use water to avoid mounting preservative replacement costs throughout a large imaging project, this practice must be avoided. Water-immersion causes osmotic shock in ethanol- or isopropyl-preserved specimens, warping specimens through shrinking or swelling, and diluting the preservative concentration in tissues (Simmons 2014). Loss in preservative strength may result in reduced antiseptic properties and specimen degradation, and the highly permeable skin of amphibians may be especially prone to the damaging forces associated with even brief exposures to a water bath.

#### Gloves

During the project, reusable flocked nitrile gloves were selected for their convenience as technicians moved between wet and dry station elements. The ability to easily don and doff wet gloves and keep hands dry to interact with the camera and computer components was essential for protecting electronics from the damaging effects of alcohol.

#### Coated forceps

Silicon-tipped forceps were used to prevent scratches in the bottom of the tank glass while positioning specimens and tags. Unprotected metal tools were avoided due to their incompatibility with the phototank.

#### Positioning and supports

Specimens prepared in non-standard poses or those not square to the camera lens when placed in the tank were gently overlain with a piece of glass to correct the plane of the body, tail, or limbs. Glass plates in standard picture glazing dimensions were stocked to provide multiple fit options to fully cover variably sized specimens. Specimens or specimens with appendages at oblique angles to the camera were propped up or stabilized with a small amount of Museum Wax (manufactured by Quakehold!).

#### Cleaning tools

A paintbrush was used for popping bubbles after specimen placement in the tank as well as for removing small fibers or scales from the bath and gently positioning tags. Surface film or cloudy blooms were siphoned out of the aquarium with a bulb syringe. Spot-removing dirt and debris with these tools extended the interval between full tank cleanings and preservative replacement.

### ﻿Computer software and technical accessories

#### Tethering cords and software

Tethering cords and software link the camera to a computer and enable remote operation. While focus stack photography is possible without tethering, this process is more time-efficient for mass digitization projects. Additionally, tethering supports better-quality images through minimizing vibrations from touching the camera, automated rotation of the focus ring and precise focal steps between shots, and large-format visualization of the stage and image details on a computer monitor so that adjustments and corrections can be made in real time. Remote operation also protects the camera from needless repeat handling and enables direct image file transfers to the desired computer or hard drive storage system, eliminating manual downloads from a memory card. Helicon Remote software was selected (https://www.heliconsoft.com/heliconsoft-products/helicon-remote/) for tethering, however, other software products such as Canon EOS Utility (Canon), Nikon Camera Control Pro (Nikon), or other brand-specific applications are all capable of remote functionality, live shooting from a computer, and digital file transfers.

#### Image stacking software

There are many commercial focus stacking software tools in use by the museum community, including Helicon Focus (https://www.heliconsoft.com/heliconsoft-products/helicon-focus/) and Zerene Stacker (http://www.zerenesystems.com/cms/stacker), which have been found to perform equally well (Brecko et al. 2014). These programs offer various methods for combining image stacks, built-in retouching tools, batch workflows, image naming and export options, and plugin integrations with Adobe Lightroom. Helicon Focus was used to carry out oMeso project digitization.

#### Image processing software

Adobe Lightroom (https://www.adobe.com/products/photoshop-lightroom.html) was used for cropping, calibrating, retouching, adding image metadata, and exporting different file formats, and was selected for the project due to its integration with Helicon Focus.

#### Power adapter

A power adapter was used as a practical accessory and is recommended for iterative imaging projects to enable the camera to run off electricity, eliminating the need to replace batteries while continuously shooting or conducting full-day imaging sessions. Power supply kit options are specific to camera system and should be vetted for safety features that ensure proper camera performance such as power surge and short circuit protection.

## ﻿Workflow

### ﻿Setup

**Cleaning**

Minimizing dirt, dust, and lint on photo station components is vital for an efficient digitization pipeline and results in less post-processing time spent on image editing (Brecko and Mathys 2020). The acrylic backdrop and camera optics were dusted immediately prior to imaging sessions, and when not in use, the photo immersion tank remained covered, and the lens cap affixed to the camera. A small container filled with 70% ethanol was used to gently dip each specimen in a pre-imaging bath before placement into the photo immersion tank. This action rinsed loose debris and molting scales present that could contaminate the phototank, ultimately extending the longevity of the imaging bath before cleaning and replacement were required.

#### Positioning

**Figure 5.**
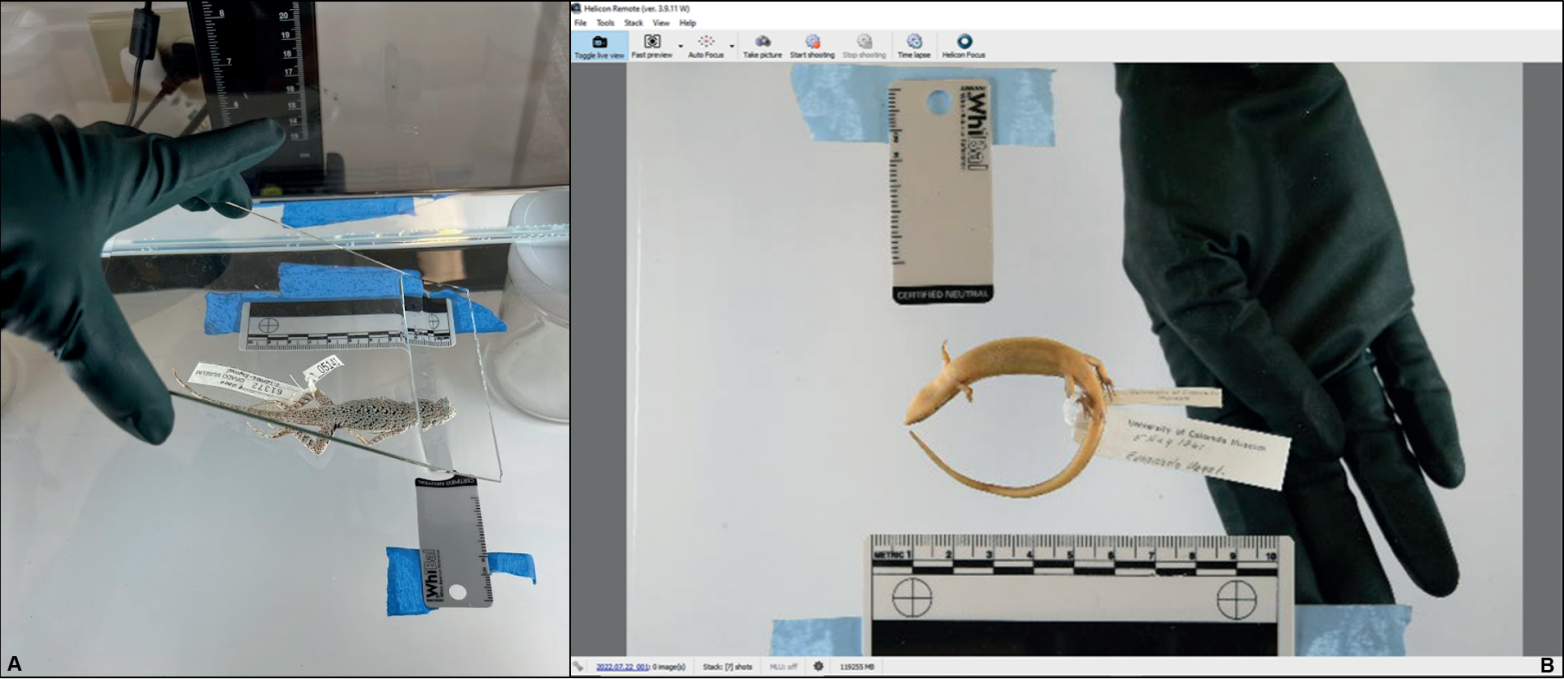
Positioning techniques. **A** a glass plate is used to gently flatten a twisted tag prior to imaging (specimen UCM 61372*Umaparaphygas*). Glass is undetectable in final images **B** the specimen (UCM 24543*Scincellaassataassata*) is positioned in the frame using the Helicon Remote ‘Live View’ function, and the scale bar and white balance card taped to the bottom of the tank are adjusted to closely border its body shape. These standards may be cropped out of final images if desired.

Each specimen was first placed dorsal side up in the tank in a left-facing orientation with nose pointed towards the zero-end of the ruler, which is consistent with widely practiced museum imaging conventions. For limbed taxa, the main axis of the body was aligned parallel to the scale bar located along the bottom edge of the tank (Fig. 5B). Snakes or other coiled taxa in non-linear formats were imaged with the head anchored at one of the major clock-bearing positions (e.g., 12 o’clock, 9 o’clock). Poorly prepared specimens or those with contorted anatomy were overlaid with a glass plate to arrange the body or tags to lie flat in one plane (Fig. 5A). This plate was large enough to fully span the field of view so that its edges were undetectable in images. If necessary, Museum Wax was occasionally applied to prop up specimens or appendages in square alignment with the camera. Tags were arranged with coated forceps or a paintbrush to extend away from the specimen and avoid overlap or obscuring of any body elements, and when possible, straightened from oblique angles so that label text remained legible in images. The exposed label surface was noted so that when the specimen was subsequently imaged from the ventral aspect, the tag was likewise rotated to capture both recto and verso label text.

#### Framing and final staging

The composition was then previewed on a computer monitor using the Live View function in Helicon Remote to fine-tune specimen position. The calibration tools affixed to the underside of the tank were adjusted to the body size of the subject, closely bordering the specimen but allowing adequate distance so that they could be cropped out of final images if desired (Fig. 5). During this step, the scale bar was placed along the base of the field of view, with the white balance card either positioned in line with the ruler or in the right or left upper corners of the frame. The camera height was then adjusted on the rail until the subject filled roughly 80% of the frame, leaving sufficient negative space surrounding the specimen to prevent body elements from approaching the edges of the composition. Finally, the field of view was spot cleaned as needed using a paintbrush and/or bulb syringe. This step was especially important for removing bubbles and debris touching or floating directly above the specimen. While unwanted noise in the image background may be later remedied using digital touchup tools, impurities physically overlapping with the specimen and obscuring anatomy cannot be removed without disrupting image authenticity.

### ﻿Imaging

#### Camera settings

**Figure 6.**
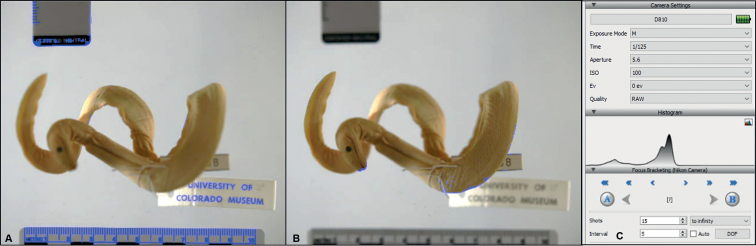
Setting focus bracket parameters in Helicon Focus. Blue highlights convey **A** the furthest distance points from the camera lens and **B** the nearest values, which are used to program the number of shots and step interval necessary to image the specimen when calculated with the specified aperture and focal length of the lens **C** the display panel shows the camera settings used to capture this Yellow-bellied sea snake (UCM 58908*Hydrophisplaturus*) and the centered exposure histogram.

Camera settings vary depending on lighting conditions and specific photo station configuration. The following parameters were used for the oMeso project and provide a good starting point when working with fluid-preserved specimens. The camera was set to manual exposure mode in order to maintain control of shutter speed, aperture, and ISO setting. A low ISO of 100 was used to prevent grainy images, as increasing this value introduces unnecessary noise that may compromise image quality. With a static subject and continuous lighting, shutter speed need not be particularly fast (e.g., 1/5–1/200 s) and should be adjusted in tandem with the aperture to achieve a balanced exposure. Because Z-stacking methods generate depth in images, it is not necessary to use a small aperture to capture a large depth of field as with single shot subject photography (generally f-stop values ≥ f/11). Rather, sharpness of the region of interest within each focal plane was prioritized over deep focus. The Helicon Remote manual suggests using the sharpest aperture supported by the lens model, which is generally two stops above its widest aperture (e.g., a lens with a maximum aperture of f/2.8 would be set to f/5.6), and this guideline was successfully applied to project specimens. Some experimentation with changing the aperture to f/11 for specimens with relatively flat profiles, such as fence lizards (*Sceloporus*) yielded satisfactory results, ultimately necessitating capture of fewer source images given the greater depth of field afforded by the setting. However, the risk of diffraction and blurred areas within images increases when narrowing the aperture, and therefore, a conservative protocol of consistently using a wider aperture (e.g., f/5.6) and more photographs in the stack to reliably produce high-fidelity images was implemented. This saved project technicians from the burden of constantly adjusting camera settings between specimens. Finally, a “fast preview” trial shot in Helicon Remote was taken prior to photo capture of each specimen in order to interpret the exposure histogram displayed by the software, as the Live View interface may not accurately reflect the exposure settings. A peak in the middle of the exposure histogram (Fig. 6) or even slightly left of center (underexposed) is ideal, ultimately granting more flexibility during image processing than an overexposed image. If the histogram showed either exposure extreme, the shutter speed and aperture parameters were adjusted until the histogram was centered, or the intensity of the light source changed by altering the directionality or distance of the lighting units from the tank. Lighting remained consistent throughout image capture to produce the best results during the stacking process.

#### Focus bracketing

Focus bracketing refers to setting focal distance steps within a scene, such as one shot focused on the foreground and others on the midground and background. When photographing natural history subjects, each source image will contain at least one part of specimen anatomy sharply in focus, ultimately creating a seamless mosaic of crisply rendered structures in the merged extended focus photo. Programming focus brackets involved indicating the nearest focusing point from the camera lens in the frame (e.g., the apex of a specimen’s back or carapace, or the caudal end of a twisting tail extending upwards towards the camera), and the furthest focusing point (generally the plane of contact between the specimen and bottom of the tank, or the calibration tools affixed underneath the tank; Fig. 6). In Helicon Focus, the distance interval between shots is automatically generated based on a combination of the specified nearest and furthest endpoints, the aperture, and focal length of the lens. A depth of field calculator is also available to ensure that the step interval provides a zone of overlap between images so that no focus band gaps (blurred areas) occur in the rendered composition. The majority of amphibians and squamate project specimens were adequately captured with 15–25 source images.

#### Rendering

**Figure 7.**
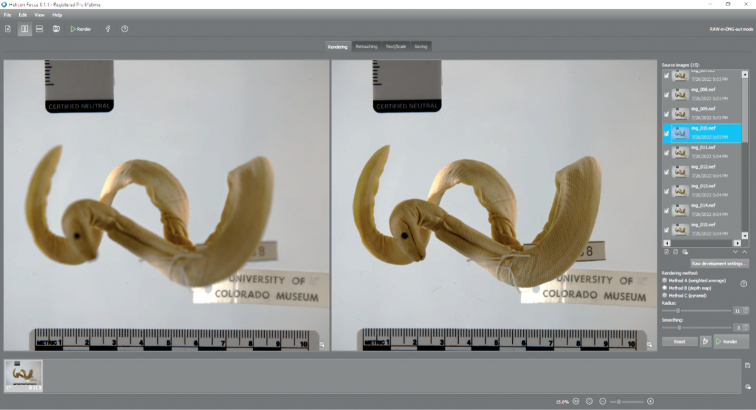
Helicon Focus interface. The left pane displays a selected source image (UCM 58908*Hydrophisplaturus*) in the stack with only the upper midbody in focus. The right view shows the fully focused output image that was rendered using Method B (depth map) to combine all 20 source images.

Following capture, the image stack was aligned using an algorithm to combine the source images (Fig. 7). The three rendering methods available in Helicon Focus include a weighted average (Method A), depth mapping (Method B), and a pyramid formula (Method C), with the first two methods working best with herpetology specimens (pers. obs.), and Method B the preferred option for rendering oMeso project specimens. While scheduling batch process jobs to run overnight in Helicon Focus is an option, it was found to be most efficient to proceed with the rendering step in real time while each specimen was still positioned in the tank. This way, a specimen could be easily reimaged should any areas in the output photo exhibit blurriness or an obscured feature from tank micro-debris without going through another **Setup** step. For this reason, imaging technicians performed a critical quality check using the magnifying glass tool immediately following rendering to ensure that all regions of the composition were in focus and satisfactory. Changes in surface depth around the margins of the specimen are especially prone to diffraction, particularly when limb elements are in relaxed positions hanging below the body plane, or with highly dimensional structures, such as the horns and modified scales in horned lizards (*Phrynosoma*), ridged tail annuli in spiny-tailed lizards (*Saara* and *Uromastyx*), and the stacked coils of preserved snakes. Blurred regions were most often remedied by adding more images to the stack to reduce the step interval, but if problem-areas persisted, the nearest and furthest focus bracketing parameters were adjusted.

#### Retouching

If necessary, the output image background was retouched prior to export from Helicon Focus (alternately, edits were applied at a later point in the workflow using image processing software). The Blurring Brush was used to clean up dirt flecks, bubbles or other alignment artifacts that trail through the background of the composite image due to the stacking procedure. Brush Hardiness and Color Tolerance settings were adjusted to seamlessly blend the background and remove particle interlopers (typically 40% and 75%, respectively), while carefully avoiding inadvertent editing of specimen anatomy. Though not employed for the oMeso project, the Dust Mapping feature is another option to remove known scratches or blemishes on the bottom of the tank or dust on the lens optics.

#### Export format

Composite images were exported as digital negative files (DNG). Like tagged image file format files (TIFF), DNG is a lossless, standardized, backward-compatible universal file format that meets best practice recommendations for archiving digital images (ADRI 2020; Corrado and Sandy 2017). Raw image formats (RAW) outputted from the camera are a proprietary lossless format that vary by manufacturer and cannot be edited by third-party software. From a digital asset management perspective, RAW is considered a less sustainable format than DNG or TIFF as there exists a greater risk of access failure and information loss over time as files become unreadable or software unsupported. Therefore, RAW formats were not maintained. During export, project images were renamed by concatenating institutional catalog number with scientific name and aspect (e.g., UCM_HERP_31447_Crotalus_lorenzoensis_dorsal.dng). Application of a standard file naming convention is highly recommended for large digitization projects for easy and intuitive file retrieval.

#### Rotate and repeat

Following dorsal image capture, each specimen was rotated to a ventral view (or opposite aspect for non-standard preparations) and the **Setup** and **Imaging** phases repeated. During rotation, the nose remained pointed towards the zero-end of the scale bar rather than flipped along the horizontal axis to ultimately generate paired images that portray both specimen aspects in the same orientation. At this point in the workflow, technicians opted to either proceed to the next step (**Image Processing**), or continue to batch capture specimens, consolidating imaging tasks and amassing several output media before shifting to photo editing work.

### ﻿Image processing

#### Photo editing

Composite output images were processed using Adobe Lightroom. In Lightroom, edits are saved as a set of instructions to a catalog file (.lrcat) instead of written directly to images, thereby preserving archival DNG/TIFF formats. While image processing is a necessary workflow step, many journals will not accept images that have been modified in ways other than whole-image manipulations (Cromey 2010). Some authors also stress that original RAW or DNG files should be made available to taxonomists for comparison to avoid doubts regarding authenticity (Aguiar et al. 2017; García-Melo et al. 2019). As such, processing steps for the oMeso project were limited to basic edits such as cropping and white balance adjustments. Photographs were first cropped to frame the specimen and calibration tools. Specimens imaged on the same day under the same lighting conditions with no modifications to studio light position were white balanced in batch. If necessary, the Lightroom Spot Removal tool was used to clean up any background blemishes not already retouched in Helicon Focus. Because focus stacking can result in darker images (Geiger 2013; Brecko and Mathys 2020), the exposure level was occasionally brightened, especially for specimens with dark coloration. This step was limited to Joint Photographic Experts Group (JPEG) image versions to ensure that online media are legible to web users, while all other imager versions are maintained without this adjustment (the processed large format TIFF and the original archival DNG, see section below), which is ultimately left to the discretion of researchers or other end users.

#### Metadata, file format specifications, and data management

Basic Exchangeable Image File Format (EXIF) metadata were packaged and added to processed images using a preset in Lightroom to inform end users of image properties. These included: institution, image technician and date, copyright, image licensing, and Creative Commons attribution requirements (https://creativecommons.org/licenses/by-nc-sa/4.0/). UCM ultimately maintains three versions of each image: the original composite DNG without edits, a processed TIFF file that meets publication criteria, and a processed JPEG. High-resolution TIFFs (300 ppi, no compression) are intended for inclusion in publications, exhibits, or digital loans, and serve as a processed large format archival version of each image. Web-accessible JPEGs (long edge set to 3500 pixels, resolution 72 ppi) have a compressed file size and are therefore more easily distributed and accessed online. Despite down-sampling, JPEGs produced using the combined focus stack and phototank setup are incredibly detailed and likely meet the needs of most stakeholders and applications. As a best practice against catastrophic data loss, three copies of each image file are maintained in different storage locations (an external hard drive, a local peta-storage architecture at the University of Colorado, and dedicated Arctos database servers at the Texas Advanced Computing System), and regularly backed up.

## ﻿Discussion

### ﻿Challenges

The time-intensive nature of this methodology may be perceived as a major limitation. Tank preparation, specimen setup, and paired dorsal and ventral image capture and processing ranges from 18–45 min per specimen. This range does not include other associated digitalization tasks such as specimen selection, project tracking, or linking images with database records and/or publishing media to biodiversity data portals. While many specimens require only minor adjustments and cleaning of the stage when placed in the tank, those in non-standard positions may extend setup times as technicians must carefully manipulate and prop anatomy to achieve the most standard view. Similarly, an ethanol bath that is approaching its expiration will extend workflow timelines given the need to edit out accumulated tank debris from images. After setting focus brackets, image capture for a stack of 20 source images runs for ca. 1.75 min. Rendering time depends both on the number of images captured in the stack as well as the processing power of the computer used, with project specimens averaging less than a minute on a Dell Intel Core i7-10700 computer. Quality checking, and image retouching and processing generally ranges from 4–10 min per photo, with overall daily project outputs averaging nine specimens, or a total of 18 processed images (including accompanying file format versions).

While a high-throughput solution does not currently exist, there are some points of efficiency in carrying out a large-scale digitization project using a wet setup. Batching specimens together of the same type and size, such as ‘small frogs less than 6 cm’ or ‘coiled snakes stored in gallon jars’, has the effect of minimizing adjustments in camera height and calibration tool placement between specimens. Avoiding the use of a larger aquarium than is necessary to accommodate target specimen body size also optimizes the pipeline, as changing out used ethanol is costly and time-consuming, and maintaining additional preservative volume only compounds these issues. However, it is important to avoid delayed replacement of dirty solution, as there are diminishing returns if technicians are investing significant time in spot-cleaning the tank during the specimen **Setup** phase or intensively editing out numerous particles and loose scales appearing in output images. This issue also underscores the post-processing time savings of maintaining the lens optics, tank, and background environments clean through covering and/or dusting equipment before each work session.

**Figure 8.**
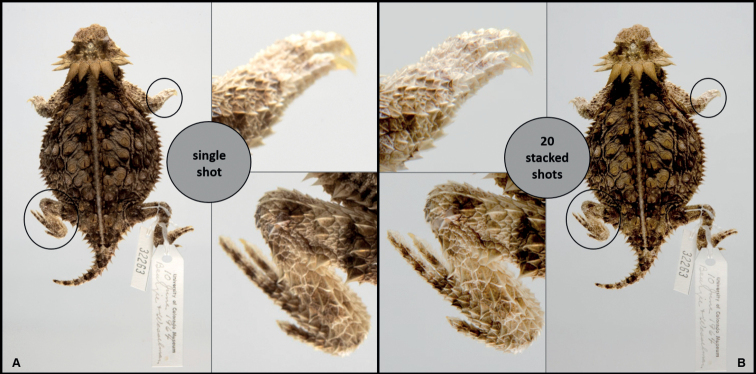
UCM 32263*Phrynosomasolare* imaged with one shot and 20 stacked images. **A** the single shot was photographed using a narrow aperture (f/13) to maximize depth of field, however, extremities and other regions with changes in depth (such as nose and tail) are out of focus under 100% magnification. Z-stacking resolves these issues. Both images are high quality and suitable for a wide range of applications, however **B** the photo generated by focus stacking is more technically sound for research applications that require fine morphological detail.

Some institutions with limited time or budget may find that using the phototank setup alone is satisfactory for digitizing specimens. A DSLR camera produces a high-quality image with a single shot, however, morphometric, meristic, and some taxonomic applications may require images with greater depth of field to adequately extract or interpret phenotypic information (see Fig. 8). For these cases, a demand-based model may be an appropriate workaround when a larger suite of images beyond the standard dorsal and ventral body aspects are needed by end users. Without performing the Z-stacking step, institutions can slightly streamline workflows, though may need to occasionally rephotograph requested specimens to provide closeup imagery of key traits when they are obscured or out of focus with a single shot capture method. Despite these bottlenecks, the utility of wet studio focus stack photography and the potential long-term positive impacts on specimen documentation, preservation, and staff resource gains through offsetting handling and loans likely outweigh the aforementioned costs and inefficiencies. Even so, there is a clear need for scalable approaches to the mass digitization of herpetology collections - and more broadly, any fluid-preserved specimen type with highly dimensional morphology - in order to enable synoptic imaging of collections.

While not explored in this project, adding lateral aspects to the imaging workflow would increase the amount of taxonomically informative media generated for each specimen, especially for species where ocular and labial scales or lateral patterning are diagnostic and not as readily observed from a dorsal or ventral vantage. Lateral views can be accomplished with the described setup using a mounting device in the tank to support and secure specimens while positioned on their sides. Ideally, this rig would be undetectable or minimally infringe on the overall aesthetics and composition of resulting images, making lateral views more broadly appealing and usable by diverse end users. It is worth noting that a tripod-mounted camera is a viable option for capturing lateral specimen aspects through the wall of the phototank when the specimen is already positioned in the aquarium for dorsal imaging. However, this method either involves transferring the camera from the copy stand to the tripod, which is inefficient and increases the possibility of mechanical damage from mishandling or dropping photography equipment; or requires procuring a secondary camera body and lens in order to efficiently operate two points of capture, which is beyond the budget of many collections. As already mentioned, a tripod system may also introduce vibration artifacts into images due to its lesser stability.

Other challenges relate to media storage costs and sustainability. Uncompressed Z-stacked output images are relatively large (averaging 2.2MB and 87MB for JPEG and TIFF formats respectively) and are more costly to store and maintain than single shot SLR photographs, or non-SLR images from phones or point-and-shoot cameras. The storage footprint for the oMeso project currently occupies approximately 2TB for dorsal and ventral images from nearly 500 specimens (three copies of each image version [DNG/TIFF/JPEG] and one copy of the raw image stacks). However, digital storage costs trend down over time, and Z-stacking consumes far less space when compared with increasingly popular 3D image modalities such as CT scans or photogrammetry models. Another suite of issues stem from managing digital images in a long-term preservation context, and tracking image usage through time. Before embarking on a large-scale digitization project, it is essential for media generators to create long-term strategies to protect against data loss and maintain data accessibility. These include planning for multiple image backups stored in geographically distinct locations, periodic testing for file corruption and vulnerabilities, and migrating to new formats as technologies become unsupported or obsolete. Best practice recommendations for maintaining media-object associations and enabling tracking through time include minting persistent resolvable identifiers for images (e.g., DOIs, ARKs, EZIDs, UUIDs, GUIDs) and requiring citation of institutional voucher catalog numbers when using images in projects, presentations, articles, or other forms of publication (iDigBio 2013; Guralnick et al. 2015; Nelson and Ellis 2019). As more herpetology collections engage in mass-imaging, another concern is the establishment of community-wide standards (García-Melo et al. 2019; Lunghi et al. 2020). Standardization in imaging methods and tools such as backgrounds, lighting, calibration measures, equipment, and protocols will produce images that are more readily compared and evaluated. This has been an ongoing topic at many Integrated Digitized Biocollections conferences and workshops (www.idigbio.org) across natural history collection types, and more work in this realm is needed.

### ﻿Summary

The relative lack of existing herpetology specimen images published to data aggregators is a glaring gap in the biodiversity media space and likely results from the inherent challenges of blurring and glare associated with photographing fluid-preserved reptiles and amphibians. Focus stack photography paired with a phototank setup mitigates these known issues, and the resulting exceptional specimen image quality enables precise identifications, phenomic analyses, and numerous other applications for downstream end users. Already, recently generated UCM specimen images serve as some of the singular depictions available on the web for certain rare taxa (e.g., *Lepidophymalipetzi*, *Pseudoeuryceaanitae*, *Xenosaurusrackhami*), and these taxonomic-grade images have been used to remotely verify and update identifications for dozens of specimens, and train deep learning algorithms to automate scale counts in lizards (see Cheung et al. 2021). The methods presented herein are easily transferrable to any fluid-preserved vertebrate group and can be adapted for most fluid-preserved specimens using alternative mounting strategies (e.g., crustaceans, aquatic insects, fossil amber). Development of accessible specimen image archives offers a viable pathway for researchers, students, conservation managers, and the general public to further explore collections, while preserving and enhancing primary physical voucher resources. Given the limitations to loaning and accessing physical collections, leveraging digital collections when possible is critical to making biodiversity data more rapidly available at a global scale in order to open and advance manifold avenues of research, education, and unanticipated collections uses.
